# Role of Intraoperative Pathology Consultation by Imprint and Scrape Cytology in Soft Tissue Tumors and Tumor-Like Lesions

**DOI:** 10.1155/2021/6633646

**Published:** 2021-10-29

**Authors:** Rupali Gautam, Harsh Mohan, Uma Handa, Bhumika Bisht

**Affiliations:** ^1^Indian Medical Association Blood Bank, Gandhidham, Kutch, Gujarat 370201, India; ^2^Oncquest Laboratories, Paras Hospitals, Panchkula, Haryana, India; ^3^Department of Pathology, Sarai Building, Government Medical College and Hospital, Sector-32, Chandigarh 160030, India; ^4^Polo Labs, Ivy Hospital, Sector 71, S.A.S. Nagar, Mohali, Punjab 160071, India

## Abstract

Intraoperative pathologic consultation plays an essential role in therapeutic decision making, possibly avoiding under or overtreatment of the patient. Common indications for intraoperative consultation include obtaining a diagnosis in an unknown pathology, ruling out malignancy, confirming a provisional diagnosis, and assessing margin status. Fifty patients undergoing surgery for soft tissue tumors or tumor-like lesions were included in the present prospective study to evaluate the role of intraoperative pathologic consultation by imprint and scrape cytology. Careful and quick gross examination of the specimen was performed, followed by processing for imprint and scrape smears. The prepared smears were evaluated by three pathologists and the cytological diagnosis compared subsequently with final histopathological diagnosis. Intraoperative consultation was primarily requested to make or confirm preoperative diagnosis. In 44.0% cases, no previous tissue/cytological diagnosis was available. In 56.0% cases, previous pathological diagnosis was available, but the reports were inconclusive or were reported from outside our institute. The diagnostic yield of imprint smears was 24% (5 malignant, 6 benign, and 1 inconclusive), and scrape smears was 100% (10 malignant, 38 benign, and 2 inconclusive). Paraffin-embedded sections yielded diagnosis in 100% cases (11 malignant, 38 benign, and 1 nonneoplastic). Imprint smears alone were not of much help in intraoperative diagnosis. Scrape smears were found to be superior to imprint smears in terms of diagnostic yield and accuracy. Combined imprint and scrape smear cytology did not provide any advantage in intraoperative provisional tissue diagnosis in soft tissue tumors.

## 1. Introduction

Soft tissue sarcomas encompass a broad spectrum of clinically, histologically, and molecularly diverse neoplasms that share a mesenchymal origin. These neoplasms present with unique diagnostic and therapeutic challenges [1]. The incidence of soft tissue sarcomas (STS) is relatively much lower than that of carcinomas and other neoplasms, constituting less than 1% of all malignant tumors [2]. Soft tissue tumors are divided into benign, intermediate, and malignant categories. Benign soft tissue tumors have a limited capacity for autonomous growth, exhibit little tendency to invade, and have a low rate of recurrence. They are about 100 times more common than sarcomas [3]. The intermediate (borderline or low malignant potential) tumors have a high rate of local recurrence but a small risk of metastasis. Soft tissue sarcomas are locally aggressive and are capable of invasive growth, recurrence, and distant metastasis. When considering all adult STS, the most common histological types include liposarcoma, leiomyosarcoma, undifferentiated pleomorphic sarcoma, and gastrointestinal stromal tumor [4].

It is important to accurately diagnose and grade these tumors for appropriate treatment and management [5]. Prior to any treatment, preoperative diagnosis of these tumors is important. For many years, tissue biopsy was considered the gold standard [6]. Accurate diagnosis relies upon recognition of characteristic histologic and cytologic features, including architecture, stromal characteristics, vascular patterns, and dominant cytology. These features may not be represented or apparent in limited core needle biopsy or fine needle aspiration specimen [7].

Intraoperative evaluation of surgical specimens can aid rapid diagnosis and guide therapeutic decision making [8]. The most common indication for an intraoperative consultation is to obtain a diagnosis in an unknown pathology, especially to determine whether it is benign or malignant. Intraoperative consultation is also done to assess margin status, confirm diagnosis, and evaluate tumor spread [9]. Frozen section or imprint cytology can be utilized for intraoperative evaluation of soft tissue tumors [10]. Touch imprint cytology (TIC) and frozen section diagnosis are valuable intraoperative guides for the management of malignancies as they help make a prompt therapeutic decision that may prevent surgical reintervention [11]. Imprint cytology is a technique in which imprints are obtained from the freshly cut surface of fresh unfixed tissue for evaluation [12]. It has certain advantages over frozen section examination as it is a simpler procedure, requiring lesser resources and time.

In view of varying reports on intraoperative consultation by various techniques, the present study aims to assess the role of imprint and scrape cytology in establishing diagnosis of soft tissue tumors and compare the diagnostic utility of cytological smears with the final histopathological diagnosis.

## 2. Materials and Methods

The present study was carried out on 50 patients with soft tissue tumors undergoing surgery. Detailed patient history and clinical and radiological findings were recorded. The intraoperative fresh tissue specimen was submitted wrapped in a gauze piece to the pathology department. Careful and quick gross examination of the specimen was performed, which was followed by processing for imprint and/or scrape cytological examination.

Cytological smears were prepared from areas of interest (solid areas, discolored areas, and areas with different appearance such as mucoid/myxoid), avoiding areas with evident necrosis or hemorrhage. Direct imprint smears were prepared by pressing the glass slide against the cut surface of fresh specimen. For scrape smears, tumor surface was scraped with edge of a glass slide and obtained tissue smeared on a clean glass slide. Light gliding movement was done to preserve cytomorphological details. Both air-dried and wet-fixed smears were prepared. In each case, at least two smears were air-dried for May-Grunwald Giemsa (MGG) staining and one wet-fixed in 95% alcohol for hematoxylin and eosin (H&E) staining. Frozen section was not employed in any of the cases.

Imprint and scrape smears were evaluated by pathologists and the diagnostic impression communicated immediately to the operating surgeon verbally, followed by written format. Turnaround time between tissue accession and conveying of report was noted. Remnant tissue was transferred in formalin for fixation and processed for routine histopathological examination. Based on provisional diagnosis made on paraffin-embedded H&E sections, special stains and immunohistochemical stains using the standard technique were employed [13].

The imprint and scrape smears were examined by three observers for cellularity, processing artefacts, and cellular and nuclear details. The cytological diagnosis was compared with final histopathological diagnosis from the resected specimen.

Descriptive statistics and the kappa test of agreement were used for statistical analysis of the observed data. The descriptive statistics was applied on imprint and scrape smears and final histopathologic diagnosis to calculate sensitivity, specificity, diagnostic accuracy, and total predictive value of procedures. The kappa test of agreement (*k*) was applied to observations of imprint and scrape cytology to find the agreement with final histopathology. *K* value more than 0.92 was considered to give an excellent significance.

## 3. Results

Age of the patients ranged from 10 to 80 years with mean age of 40.0 ± 15.4 years. Male to female ratio was 0.8 : 1. Patients presented with complaint of swelling (54.0%) or swelling associated with pain (46.0%). The lesions were more commonly situated on trunk (30.0%), followed by lower limb (28.0%), head and neck (24.0%), and upper limb (14.0%). Most of the soft tissue lesions were superficial (70.0%). Deep-seated soft tissue lesions constituted 26.0%, and those in retroperitoneum constituted 4.0%. Out of the 38 benign tumors and one nonneoplastic lesion diagnosed on paraffin-embedded sections, 34 cases were superficially located. Out of 11 malignant cases, 7 cases were deep-seated and 2 cases were retroperitoneal in location. Clinicoradiological diagnosis was benign in 31 cases (62.0%), malignant in 15 cases (30.0%), and inconclusive in 4 cases (8.0%). Preoperative FNAC or biopsy diagnosis was available in these four cases.

Twenty-eight cases (56.0%) had a previous pathological diagnosis available in the form of FNAC or biopsy. However, intraoperative pathologic consultation was still requested to confirm the diagnosis. In 22 cases (44.0%), no previous tissue diagnosis was available.

The diagnostic yield of imprint smears was 24% (12 out of 50 cases) and scrape smears was 100% (50 out of 50 cases). The diagnostic yield of paraffin-embedded sections was 100%. Therefore, in this study, the diagnostic yield of scrape cytology was comparable to that of paraffin-embedded sections.

**Figure 1:**
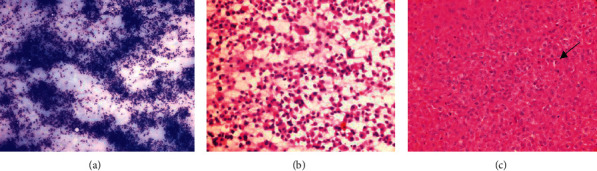
Malignant small round cell tumor (Ewing's sarcoma). (a) Imprint smear showing sheets of small round to oval tumor cells showing moderate atypia. Cytoplasm is vacuolated (H&E, X400). (b) Scrape smear showing sheets and singly scattered malignant small round tumor cells (MGG stain, X200). (c) Paraffin-embedded section showing malignant small round tumor cells arranged in sheets with coarse nuclear chromatin and scant cytoplasm. Mitotic figures are also seen (arrow) (H&E, X400).

**Figure 2:**
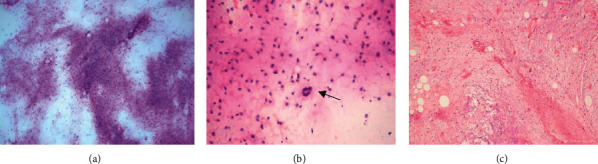
Myxoid liposarcoma. (a) Case diagnosed as atypical lipomatous tumor on scrape smear showing fragments of fibroadipose tissue and atypical cells (H&E, X100). (b) Scrape smear showing round to oval shaped tumor cells showing mild atypia. Note floret cell is seen (arrow) (H&E, X400). (c) Section showing spindle-shaped cells with hyperchromatic nuclei along with mature adipocytes. Background showing myxoid matrix (H&E, X100).

**Figure 3:**
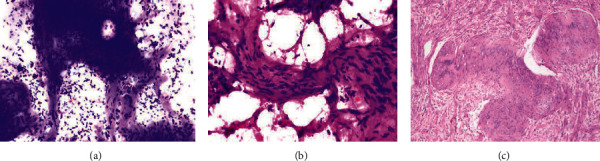
Schwannoma. (a) Imprint smear showing clusters and palisades of spindle cells (MGG stain, X100). (b) Scrape smear showing cluster of spindle cells having elongated wavy nuclei (H&E, X400). (c) Spindle-shaped tumor cells with bland nuclear chromatin seen on paraffin-embedded sections. Nuclear palisading leading to Verocay body formation noted (H&E, X200).

**Figure 4:**
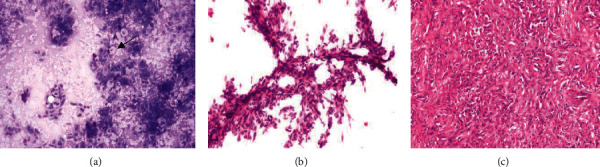
Dermatofibroma. (a) Case diagnosed on imprint smear as benign spindle cell tumor of fibrous origin showing clusters and singly scattered spindle-shaped cells. Background showing foamy macrophages (arrow) (MGG stain, X100). (b) Scrape smear showing cluster of spindle-shaped cells showing bland nuclear chromatin and cytoplasmic projections (H&E, X400). (c) Paraffin-embedded section showing singly scattered and fascicles of spindle-shaped tumor cells with bland nuclear chromatin (H&E, X200).

Among the 50 cases diagnosed on paraffin-embedded sections (considered as gold standard in this study), 11 were malignant (22.0%), 38 were benign (76.0%), and 1 (2%) was nonneoplastic. On imprint smears, diagnosis of malignant neoplasm was given in 5 cases (10%), benign neoplasm in 6 cases (12%), and one case was regarded as inconclusive (2.0%). On scrape cytology, 10 cases (20.0%) were diagnosed as malignant neoplasms, 38 cases (76.0%) as benign neoplasms, and 2 cases (4.0%) as inconclusive. The diagnostic accuracy of scrape smears was 97.8%. Combined imprint and scrape smears were diagnostic in 48 (96.0%) out of 50 cases, while remaining 2 cases (4.0%) were inconclusive. The diagnoses given by each technique and their correlation with paraffin-embedded sections are given in Table 1. A few representative examples on tissue pathology are shown in Figures 1–4. Among malignant neoplasms, Ewing's sarcoma and liposarcoma were the most frequent. Benign tumors were mostly lipomatous tumors.

The concordance rate between diagnoses of imprint and scrape cytology and paraffin-embedded sections was 86.0% (43 out of 50 cases). Nine samples diagnosed as malignant neoplasm on imprint and scrape cytology were confirmed as malignant on paraffin-embedded sections. However, type-specific diagnosis was possible in only 8 cases (88.8%). Among benign category, scrape smears diagnosed all lipomatous tumors except one which was labelled as benign spindle cell tumor of neural origin.

There was one false-negative case on scrape cytology (2.6%). It was reported as inconclusive due to limited cellularity and diagnosed as spindle cell variant of liposarcoma on histopathologic examination. No false-positive result was seen.

The statistical result of scrape smears for distinguishing malignant from benign lesions is given in Table 2. The kappa test of agreement scrape smear and paraffin-embedded section for making diagnosis (*k* = 0.93) gave good significance (*k* > 0.92).

## 4. Discussion

Management of soft tissue tumors requires correct diagnosis, which includes identification of benign/malignant nature of tumor, histologic subtype, and degree of differentiation. With passing decades, significant improvement has been observed in this field owing to a better imaging technique, effective chemotherapy, and increasing use of limb-salvage surgery. A proper pathological examination of the biopsy specimen substantiated by immunohistochemical markers is essential in reaching an accurate diagnosis.

In this study, age of the patients ranged from 10 to 80 years with mean age of 40.0 ± 15.4 years. Most patients were in the age group of 30–39 years (31.4%). Dutta et al. reported slightly lower mean age of patients (35.6 years) with a range of 4 months to 80 years [14]. However, in another study by Colletti et al., a slightly higher mean age of patients (59.9 years) with a range of 12–95 years was reported [15]. The ratio of male to female patients was 0.8 : 1 in this study. The ratio of male to female patients was slightly higher (1.05 : 1) in the study by Dutta et al. [14].

The reason for an intraoperative pathologic consultation in this study was to establish or confirm tissue diagnosis (50 cases). In 23 cases (46.0%), no previous tissue diagnosis was available. In the twenty-eight cases (56.0%) where previous pathological diagnosis was available, intraoperative pathologic consultation was only done for confirming diagnosis. In the study by Suen et al. [16], the major clinical reason for obtaining intraoperative consultation was rapid tissue diagnosis. Two studies (Ranjan et al. [17] and Khalid et al. [10]) showed reason for intraoperative pathologic consultation as making tissue diagnosis only.

Imprint cytology has been demonstrated to be an excellent diagnostic tool with high sensitivity and specificity in comparison to frozen section. Eventhough architectural orientation is better appreciated in case of frozen sections, artifacts are more commonly encountered. Morphological details are more vivid in imprint smears. The turnaround time is also less in case of imprints [18].

In the current study, the diagnostic yield of imprint smears was 24% and scrape smears was 100%. The diagnostic accuracy in distinguishing benign from malignant lesions by imprint smears was 22.0% and scrape smears was 97.8%. Malignant small round cell tumor was the most common malignant neoplasm diagnosed by imprint and scrape smears. Among the benign category, schwannoma was the most common neoplasm diagnosed by imprint smears. Lipoma was the most frequent benign tumor diagnosed by scrape smears followed by schwannoma.

Although the role of imprint and scrape cytology has been studied in epithelial tumors, there is no study in literature that analyses the combined role of imprint and scrape cytology on soft tissue tumor diagnosis. The diagnostic accuracy obtained in this study parallels the diagnostic accuracies obtained in studies by Khalid et al. [10], in which 10% of the total cases were soft tissue tumors, and Scucchi et al. [19], which included 13% cases of soft tissue tumors. The reason for inability to arrive at diagnosis in both these studies was hypocellularity as also in the present study. In another study by Tamhane et al. [11], tissue imprint cytology (TIC) did not give satisfactory cell yield, and false-negative rates of TIC were significant as cell sampling was not satisfactory. In the study by Dutta et al. [14], inadequate imprint smears were obtained in 12.2% of soft tissue tumor cases. The probable reasons for inadequacy were highly cohesive tumor cells, increased fibrosis or sclerosis, and excessive necrosis.

The concordance rate between diagnosis of combined imprint and scrape cytology and paraffin-embedded sections in the present study was 86.0%. A higher concordance rate of 96% was observed in another study [19]. False-negative case accounted for 2.6% cases in our study. The reasons for misinterpretation were scanty cellularity or sampling error. Sampling error was faced in diagnosing pigmented villonodular synovitis, angiomyxolipoma, malignant peripheral nerve sheath tumor, and liposarcoma. Suen et al. [16] found 6% and Dutta et al. [14] reported 12.2% false-negative cases due to similar reasons.

The specificity (100%), positive predictive value (100%), negative predictive value (97.8%), and diagnostic accuracy (97.8%) reported in the present study compare well with those observed in few other studies [10, 19].

In terms of tumor typing, imprint and scrape smears correctly diagnosed nine malignant tumors. However, type-specific diagnosis was possible in 90.9% cases, which is less than the diagnostic accuracy (97.3%) observed in another study [20]. In the study by Dutta et al. [14], the accuracy of imprint smears for diagnosis of both benign and malignant soft tissue tumors was 75%. The imprint and scrape smears in the present study gave three diagnoses as malignant small round cell tumor, which were labelled as Ewing's sarcoma on final paraffin sections. The subtyping of sarcoma cases was difficult. The reason could be sampling issues. Cherie et al. [21] found similar difficulties while providing provisional diagnosis in some specific malignancies on tissue imprints. In the benign neoplastic group, diagnosis of lipomatous tumors was given in 68.4% cases. Subtyping of lipomatous tumors was done accurately in all cases except two cases (5.2%).

In the present study, the diagnostic yield of scrape smears was 100.0%. Out of 50 cases diagnosed on paraffin-embedded sections, 43 cases (86.0%) were accurately diagnosed on imprint and scrape cytology. Two cases (4.0%) had inconclusive diagnoses due to interpretative and sampling errors. High diagnostic yield (97.3%) has also been reported by Kotle et al. [20]. Their study included 10 cases of soft tissue tumors, out of which nine cases (90.0%) were correctly diagnosed on imprint cytology. In a study be Suen et al. [16], which included 37 cases of soft tissue tumors, 31 cases (83.7%) were accurately diagnosed on imprint cytology. Dutta et al. [14] accurately diagnosed 75% cases of soft tissue tumors by imprint smears. Higher concordance was observed in a study by Bui et al. [8], where imprint cytology was performed on 160 cases of bone and soft tissue tumors, and it yielded accurate diagnosis in 156 cases (98.0%).

The provision of diagnosis by imprint and scrape cytology to the operating surgeon helped confirm preoperative diagnosis made on clinic-radiological findings. Among malignant cases, one case with a preoperative diagnosis of nerve sheath tumor was reported as benign spindle cell tumor of neural origin on imprint and scape cytology and MPNST developing in plexiform neurofibroma on final histopathology. This case had undergone wide local excision due to large size and clinical assessment of margins. Second case had a preoperative diagnosis of sarcoma, reported on imprint and scrape cytology as nerve sheath tumor with possibilities of schwannoma and MPNST. Final histopathological diagnosis was synovial sarcoma. Though there was discordance in typing, this case had also underdone the standard surgical management with wide local excision. There was a suspected case of retroperitoneal sarcoma that was inconclusive on imprint and scrape cytology due to insufficient cellularity. Final histopathological diagnosis was spindle cell variant of liposarcoma. All these cases had clear margins on histopathology.

## 5. Conclusion

The present study indicates that imprint smears alone were not of much help in providing intraoperative diagnosis for soft tissue tumors. Scrape smears were found to be superior to imprint smears in terms of diagnostic yield and accuracy. Combining imprint cytology with scrape smear cytology for intraoperative diagnosis did not offer any advantage over scrape cytology alone and has higher accuracy, sensitivity, and specificity. Scrape cytology alone can serve as an efficient and reliable diagnostic tool for intraoperative evaluation of soft tissue tumors if representative sampling is ensured. However, the present series is limited to a small sample group, especially in terms of malignant soft tissue tumors. Valid conclusions and recommendations require further studies in large series.

## Figures and Tables

**Table 1:** Comparison of imprint and scrape cytology and final histopathology.

Imprint cytology	Scrape cytology	Histopathology
Malignant neoplasms, *n* *=* **5 (10.0%)**	*n* = **10 (20.8%)**	*n* = **11 (22.0%)**
MSRCT (*n* = 3)	MSRCT (*n* = 3)	Ewing's sarcoma (*n* = 3)
Sarcoma, NOS (*n* = 1)	Sarcoma closest to myxoid liposarcoma (*n* = 1)	Liposarcoma, well differentiated (*n* = 1)
	Atypical lipomatous tumor likely liposarcoma (*n* = 1)	Liposarcoma, myxoid type- low grade (*n* = 1)
		Liposarcoma, spindle cell variant (*n* = 1)

Pleomorphic sarcoma (*n* = 1)	Synovial sarcoma (*n* = 1)	Synovial sarcoma (*n* = 2)
	MPNST (*n* = 1)	
	Sarcoma NOS (*n* = 1)	Sarcoma, myxoid type (*n* = 1)
	Pleomorphic sarcoma (*n* = 1)	Undifferentiated high grade sarcoma (*n* = 1)
	MPNST (*n* = 1)	MPNST (*n* = 1)

Benign neoplasms, *n* = **6 (12.0%)**	*n* = **38 (79.1%)**	*n* = **38 (79.1%)**
Schwannoma (*n* = 2)	Consistent with lipoma (*n* = 22)	Lipoma (*n* = 22)
	Consistent with lipoma (*n* = 1)	Angiolipoma (*n* = 1)
	Consistent with lipoma (*n* = 2)	Fibroepithelial polyp (lipofibroma) (*n* = 2)
	Benign spindle cell tumor of neural origin (*n* = 1)	Angiomyxolipoma (*n* = 1)

Benign spindle cell tumor of fibrous origin (*n* = 1)	Schwannoma (*n* = 6)	Schwannoma(*n* = 7)
	Benign spindle cell tumor possibly of fibrous origin (*n* = 1)	

Benign spindle cell tumor of neural origin (*n* = 1)	Benign spindle cell tumor of fibrous origin (*n* = 1)	Deep fibromatosis (desmoid) (*n* = 1)
Benign spindle cell lesion (*n* = 1)		Dermatofibroma (*n* = 1)
Ganglion (*n* = 1)		Pigmented villonodular synovitis (*n* = 1)
		Consistent with ganglion cyst (*n* = 1)
		Organizing hematoma (*n* = 1)

Inconclusive, *n* *=* **1 (2.0%)**	*n* = **2 (4.0%)**	
Descriptive (*n* = 1)	Descriptive (*n* = 2)	

Nonneoplastic lesion		*n* = 1 (2.0%)
		Organizing hematoma (*n* = 1)

Bold values represent the number of cases which were malignant, benign, nonneoplastic, and inconclusive out of total 50 cases of soft tissue tumors which were processed for imprint and scrape cytology and later compared with final histopathology.

**Table 2:** Statistical analyses of results given by the scrape smear technique.

Sensitivity (%)	Specificity (%)	Positive predictive value (%)	Negative predictive value (%)	Diagnostic accuracy (%)	Kappa test of agreement
90.0	100	100	97.4	97.8	0.92

## Data Availability

The data used to support the findings of this study are included within the article.
